# Genome-Wide Identification of the Q-type C2H2 Transcription Factor Family in Alfalfa (*Medicago sativa*) and Expression Analysis under Different Abiotic Stresses

**DOI:** 10.3390/genes12121906

**Published:** 2021-11-27

**Authors:** Jun Pu, Mingyu Li, Pei Mao, Qiang Zhou, Wenxian Liu, Zhipeng Liu

**Affiliations:** State Key Laboratory of Grassland Agro-ecosystems, Key Laboratory of Grassland Livestock Industry Innovation, Ministry of Agriculture and Rural Affairs, Engineering Research Center of Grassland Industry, Ministry of Education, College of Pastoral Agriculture Science and Technology, Lanzhou University, Lanzhou 730020, China; puj21@lzu.edu.cn (J.P.); limy19@lzu.edu.cn (M.L.); maop17@lzu.edu.cn (P.M.); zhouq2013@lzu.edu.cn (Q.Z.); liuwx@lzu.edu.cn (W.L.)

**Keywords:** alfalfa, Q-type C2H2 zinc finger protein family, phylogenetic analysis, abiotic stress, expression pattern

## Abstract

Q-type C2H2 zinc-finger protein (C2H2-ZFP) transcription factors are associated with many plant growth development and environmental stress responses. To date, there have been few analyses of the Q-type *C2H2-ZFP* gene family in alfalfa (*Medicago sativa* subsp. *sativa*). In this study, we identified 58 Q-type *C2H2-ZFP*s across the entire alfalfa genome, and the gene structure, motif composition, chromosomal mapping, and *cis*-regulatory elements were explored, as well as the expression profiles of specific tissues and the response under different abiotic stresses. According to their phylogenetic features, these 58 *MsZFP*s were divided into 12 subgroups. Synteny analysis showed that duplication events play a vital role in the expansion of the *MsZFP* gene family. The collinearity results showed that a total of 26 and 42 of the 58 *MsZFP* genes were homologous with *Arabidopsis* and *M. truncatula*, respectively. The expression profiles showed that *C2H2-ZFP* genes played various roles in different tissues and abiotic stresses. The results of subsequent quantitative real-time polymerase chain reaction (qRT-PCR) showed that the nine selected *MsZFP* genes were rapidly induced under different abiotic stresses, indicating that C2H2-ZFP genes are closely related to abiotic stress. This study provides results on *MsZFP* genes, their response to various abiotic stresses, and new information on the C2H2 family in alfalfa.

## 1. Introduction 

Zinc finger proteins (ZFPs) are one of the largest families of transcription regulators, with a highly conserved domain; they play crucial roles in plant development and abiotic stress responses in natural plants [1]. According to their different residues, zinc finger proteins are divided into several types, including C2H2, C2HC, C2HC5, and other [2]. Among these types, C2H2 ZFPs are the most numerous in eukaryotes [3]; they contain two cysteine (Cys2) and two histidine (His2) residues. In order to ensure the stability of their three-dimensional structure, a zinc ion is coordinated by Cys2/His2 (C2H2) with a ββα fold [4]. 

The Q-type ZFPs are a subfamily of the C2H2 ZFPs with a conserved “QALGGH” motif at the region of the zinc finger domain within the Cys2 and His2 residues; they participate widely in biological processes, and are endemic to plants with C2H2 ZFPs [5,6,7]. According to a previous study, the first C2H2-ZFP was found in *Petunia*, and 21 Q-type *ZFP*s were identified according to their special structure, which plays important roles in the activity of DNA binding [8]. More Q-type ZFPs have been observed in plants such as *Arabidopsis*, wheat (*Triticum aestivum*), and potato (*Solanum tuberosum*) [1,9,10]. All these Q-type *ZFP*s play vital roles in flowering time, leaf development and other biological processes, as well as response to abiotic stress [11]. For example, overexpression of *ZFP36*, an abscisic acid (ABA)-positive gene, increased tolerance to water stress and reduced competition for antioxidant enzymes in the *ZFP36* mutant, and was induced by ABA signalling in rice [12]. In transgenic *Arabidopsis*, *GmZF1* enhances tolerance during cold stress [13]. *TaZFP1* plays vital roles in mediating salt stress tolerance by affecting other physiological processes [14]. The *ZFP182* genes in rice participate in ABA-induced abiotic stress responses [15]. In addition, the overexpression of *STZa* in *Arabidopsis* displayed drought tolerance [7]. Therefore, the C2H2-ZFP gene family plays important roles in improving abiotic stress resistance in plants. Nevertheless, little research has been carried out on Q-type C2H2-ZFPs in alfalfa.

Alfalfa (*Medicago sativa* subsp. *sativa*) is one of the most economically valuable plants in the world, with high nutritional value and strong adaptability [16], famous as the “king of herbages” and widely planted in the northern area of China. However, adverse growth environments such as low temperatures, water deficit, and salinization of soil affect alfalfa survival and growth, restricting yield in the entire area. Therefore, in Northern China it is critical to cultivate and select alfalfa germplasm with stress resistance to enable better development of animal husbandry.

In the current study, we identified 58 Q-type *C2H2-ZFP* members in alfalfa. The phylogenetic relationships, genomic location, gene structure, chromosome distribution, gene duplication and *cis*-regulatory elements were also explored. In addition, we analyzed the expression profiles of *MsZFP*s in various tissues and under abiotic stress. These results will provide a valuable resource in exploring the Q-type C2H2 gene family and understanding the roles of the *MsZFP* gene family in alfalfa, especially the functional characterization of plant development processes and abiotic responses.

## 2. Materials and Methods

### 2.1. Database Sources and Identification of the C2H2 Gene Family in Alfalfa

We downloaded 211 *Arabidopsis* C2H2 ZFP sequences from The *Arabidopsis* Information Resource (TAIR) (http://www.arabidopsis.org, 25 May 2021) and 218 C2H2-ZFP sequences of *M. truncatula* from a published article [17]. All these plant C2H2 protein sequences were queries to search the alfalfa C2H2 family through local protein blast (BLASTP) against the whole genome sequence database [18], with an E-value threshold of 0.00001. In addition, redundant protein sequences were removed using the CD-Hit website (www.bioinformatics.org, accessed on 25 May 2021) with the default parameters [19]. The remaining C2H2 sequences were confirmed by Pfam (www.pfam.xfam.org, accessed on 27 May 2021) and SMART (http://smart.embl-heidelberg.de/, accessed on 16 June 2021), then Q-type C2H2-ZFP members were selected [20,21]. Subsequently, the ProtParam Tool (https://web.expasy.org/protparam/, accessed on 17 June 2021) was used to infer the index values of the grand average of hydropathicity (GRAVY) and the theoretical isoelectric point (pI) of these putative *MsZFP* genes [22]. Finally, we used the WoLF-PSORT (https://www.genscript. com/wolf-psort.html, accessed on 25 June 2021) to predict the subcellular localization of *MsZFP* [23].

### 2.2. Analysis of Phylogenetic, Gene Structure and Conserved Composition MsZFP Genes

To clarify the evolutionary relationship of these *MsZFP* genes, Clustal W software was used to align these *MsZFP* sequences with initial parameters [24], and MEGA 6.0 was used to construct a phylogenetic tree with the neighbour joining (NJ) method and 1000 replicate bootstrap tests [25]. The *MsZFP* gene structures were explored from the Gene Structure Display Server (www.gsgd.gao-lab.org, accessed on 17 July 2021). MEME v5.1.1 (http://meme-suite.org/index.html, accessed on 26 July 2021) was used to estimate their conserved motifs [26]. The selected number of motifs was determined according to a previous study [27], and the default settings were used for the other parameters.

### 2.3. Analysis of Chromosomal Distribution and Gene Duplication

Based on MapChart software, we were able to recognize the genomic mapping of *MsZFP* genes and their relative distances according to the genome annotation data of alfalfa. Gene duplication is a major source for expanding gene families and producing new genes in eukaryotes. To explore the gene duplication events of 58 *MsZFP* genes, we used TBtools software to perform a collinearity analysis [28]. In addition, Dnasp5 software was used to analyse the nonsynonymous (Ka) and synonymous (Ks) replacement rates of genes after replication, and to explore the mechanism of gene differentiation after replication [29,30].

### 2.4. Cis-Regulatory Element Analysis

The PlantCARE database (http://bioinformatics.psb.ugent.be/webtools/plantcare/html/, accessed on 7 November 2021) was used to identify the *cis*-regulation of the changed *MsZFP*s under abiotic stresses, according to the sequences of 2000 bp from the promoter region of *MsZFP* genes. We explored ten *cis*-regulatory elements related to stress, hormones, and light response, including ABRE (ABA-responsive element), ARE (antioxidant response element), CGTCA motif, DRE (dehydration responsive element), G-box, MBS (MYB binding site), LTR (low-temperature responsiveness), TC-rich repeats, TCA element, and TGACG motif. All these are involved in abscisic acid response, anaerobic induction, MeJA response, abiotic stresses, light response, drought inducibility, cold response, defence against stress, and salicylic acid response [31].

### 2.5. Tissue Specific Expression of MsZFP Genes

Based on the expression profiles of these *MsZFP* genes in specific tissues, we were able to speculate on their roles in alfalfa tissue development. Furthermore, the expression profiles of *MsZFP* genes in various tissues were explored by genome-wide transcriptome data downloaded from the CADL-Gene Expression Atlas, released by the Noble Research Atlas (https://www.alfalfatoolbox.org, accessed on 28 May 2021) for a total of six tissues of transcriptome data, including leaves, flowers, pre-elongated stems, elongated stems, roots and nodules. Subsequently, TBtools software was used to generate a heatmap of *MsZFP* gene expression data.

### 2.6. Analysis of These MsZFP Genes under Abiotic Stress

Four transcriptome sequencing projects were performed in previous studies, including cold stress (SRR7091780-SRR7091794) [32], drought, salt stress and ABA treatments (SRR7160313-SRR7160357) [33,34]. In this research, the expression of *MsZFP* genes was obtained through local protein blast (BLASTP) against these four transcriptomes, using TBtools software to generate heatmaps.

### 2.7. Plant Materials and Stress Treatments

The cultivated variety of XinJiangDaYe alfalfa was used as experimental material and cultivated by a hydroponic experiment in an artificial chamber. To ensure consistency of germination, seeds were vernalized under 4 °C conditions. After three days of germination, these seedlings were moved into specific nutritional conditions (1/2 MS, pH = 5.8) and cultivated under long-day conditions. Until the third leaf appeared, the plants were placed under different stress treatments (approximately 13 days after vernalization). For cold stress, these seedlings were cultivated in an artificial climate chamber with a 16 h light/8 h dark cycle at 4 °C. Four treatment time points (2, 6, 24, and 48 h) were sampled and used as a control. For drought and salt stress, these seedlings were put into specific nutritional conditions with 400 mM mannitol and 250 mM NaCl, respectively, and cultivated under a 16 h light/8 h dark cycle at 22 °C. A total of eight treatment time points were used (1, 3, 6, 12, 24 h, abiotic stress removal 1 and 12 h), along with CK (“CK” indicates 0 h). Under ABA stress, the seedlings were moved to a specific nutritional condition with 10 μM ABA, and a total of four time points were sampled (CK, 1, 3, and 12 h). Furthermore, the whole seedling was harvested under a cold environment, and the root tip was harvested for the ABA, drought, and salt treatments. Every sample contained six seedlings, and these samples were quick-frozen in liquid nitrogen and stored at −80 °C.

### 2.8. Quantitative Real-Time PCR Analysis

A total of nine *MsZFP*s were selected as candidates for abiotic resistance genes. With reference to the manufacturer’s instructions, the TRIzol method (Sangon Biotech, Shanghai, China) was used to extract the total RNA, in which cold stress RNA was extracted from the whole seedling and the other three stresses (ABA, drought, and salt) were extracted from the root tips of alfalfa. These RNAs were reverse-transcribed into single-stranded cDNAs according to the manufacturer’s protocol using the FastKing RT Kit with gDNase (Tiangen Biotech, Beijing, China). Subsequently, a NanoDrop ND1000 spectrophotometer (Thermo Scientific, Waltham, MA, USA) was used to determine the concentration of each sample. qRT-PCR was performed on all of the selected genes with the CFX96TM Real-Time PCR Detection System (Bio-Rad, Los Angeles, USA). Every 10 μL reaction volume consisted of 5 μL of 2 × SG Fast qPCR Master Mix, 1 μL of cDNA, 1 μL of DNF buffer, 0.2 μL of each of the forward and reverse primer at 10 μM, and 2.6 μL of sterilized ddH_2_O. The reaction process was set as follows: denaturation (95 ℃/30 s) and 40 cycles at 95 °C /5 s and 60 °C /30 s. Every reaction consisted of three replicates, and according to the expression of the *Medicago* actin gene (AA660796) [35], the relative expression level of *MsZFP* genes was normalized with the 2^−ΔΔCq^ method. According to the genome data of XinJiangDaYe alfalfa, all *MsZFP* gene primer pairs were designed using Primer 6 software; the lengths of the PCR products are listed in Table S1.

## 3. Results

### 3.1. Identification and Physicochemical Properties of MsZFPs

A total of 58 *MsZFP* genes were obtained from the alfalfa genome after screening domains and removing redundant genes, and *MsZFP01* was rewritten to *MsZFP58* based on the order of occurrence of the genome data (Table S2). In addition, the quantity of amino acids, molecular weight (MW), pI, GRAVY and subcellular localization of these MsZFP proteins were analysed (Table S3). These 58 putative MsZFP proteins ranged from 126 (*MsZFP14*) to 1633 amino acids (AA) (*MsZFP40*) in length. The MWs of these proteins were varied from 14000.01 Da in *MsZFP14* to 184189.9 Da in *MsZFP40*, and their predicted pI ranged from 4.83 (*MsZFP31*) to 10.03 (*MsZFP06*). In addition, the GRAVY of all MsZFP proteins were negative (<0), varying from −1.194 (MsZFP106) to −0.169 (MsZFP40), suggesting that they were soluble hydrophilic proteins. Moreover, all these Q-type ZFPs were nuclear-localized according to the results of subcellular localization analysis.

### 3.2. Phylogenetic Analysis of the Selected MsZFPs

**Figure 1:**
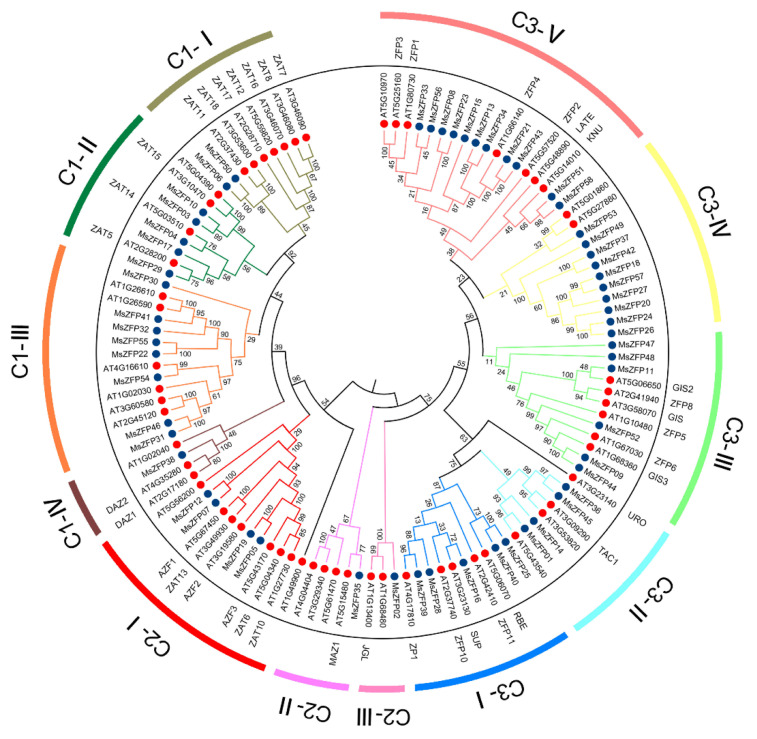
Phylogenetic analysis of Q-type *MsZFP*s; the phylogenetic tree was created using the MEGA 6.0 program (bootstrap value set at 1000 with neighbour-joining). The phylogenetic tree represents the relationship between 58 Q-type *C2H2-ZFP* genes of alfalfa and 58 Q-type *C2H2-ZFP* genes of *Arabidopsis*. All *C2H2-ZFP* genes were clustered into five clades and twelve groups, distinguished here by different colours.

To clearly explore evolutionary relationships, a phylogenetic tree was constructed with 58 Q-type *MsZFP*s and 58 *Arabidopsis ZFP*s (Table S4) by MEGA6 with the NJ method. All these *MsZFP*s were grouped into three different groups (Figure 1). Furthermore, these alfalfa *MsZFP*s were closely clustered with *Arabidopsis ZFP*s (Figure 1), and many of the functions of these *Arabidopsis ZFP*s were known, suggesting that some of these alfalfa gene functions could be inferred according to *Arabidopsis*. Based on clades and bootstrapping, the resulting phylogenetic trees were divided into twelve subclasses, including C1-Ⅰ to IV, C2-Ⅰ to III, and C3-I to V. According to these results, the same phylogenetic clades may represent the most intimate homologous gene pair of alfalfa and *Arabidopsis*. In addition, 58 *MsZFP*s were divided into different groups. Groups C1, C2, and C3 possessed 16, 6, and 36 *MsZFP*s, respectively. Furthermore, the main clades, C1 and C3, had 4 and 5 subclasses, respectively.

### 3.3. Gene Structure and Motif Composition Analysis of the MsZFPs

**Figure 2:**
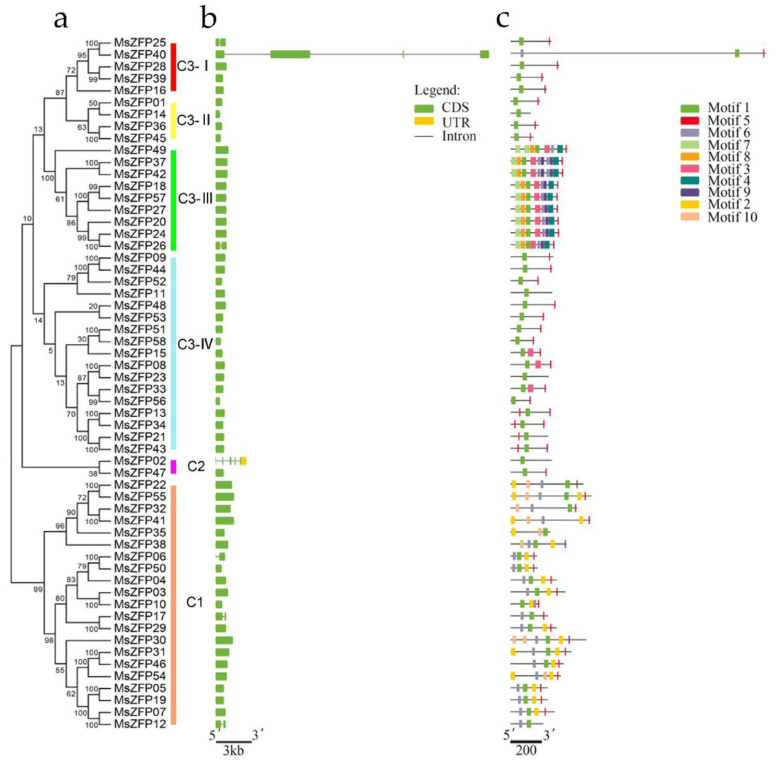
Phylogenetic relationship, gene structure, and motif compositions of alfalfa Q-type *C2H2-ZF* genes. The phylogenetic tree was constructed using MEGA 6.0 with the neighbour-joining method. (**a**) Multiple alignments of 58 protein sequences of alfalfa *MsZFP*s executed by ClustalW. (**b**) Exon/intron structures of alfalfa *C2H2-ZFP* genes. Exon/intron structures were analyzed by the Gene Structure Display Server. Exons/introns of each subgroup are represented by green boxes and black lines, respectively. (**c**) Schematic representation of the conserved motifs, as identified by MEME; each coloured box represents a motif.

We could predict the evolutionary process by analysing the gene structures. To better understand the structural diversity of *MsZFP*s, we analysed the number of exon and intron structures of all *MsZFP*s (Figure 2B). The results showed that the number of introns varied from zero to four. According to the number of introns, 50 *MsZFP*s (86.20%) were intronless, six *MsZFP*s had one intron (10.34%), and the other two *MsZFP* genes contained at least three introns.

We identified ten conserved motifs in *MsZFP*s with the MEME tool, with variable lengths from 8 to 36 amino acids (Figure 2C, Table S5). Among these motifs, motif 1 was widely discovered in all *MsZFP*s except *MsZFP41* and *MsZFP54*, matching up with the Cys-X2-Cys-X12-His-X3-His single structure. Interestingly, motif 5 was present at the C-terminal region of all the *MsZFP*s except *MsZFP13*, which is the most dominant transcriptional repression motif in plants [10]. Moreover, some motifs were specific to specific groups. For example, motifs 2 and 10 existed only in Group C1. Motifs 4, 8, and 9 were unique to clade C3-Ⅲ.

### 3.4. Chromosomal Distributions and Gene Duplications in MsZFPs

**Figure 3:**
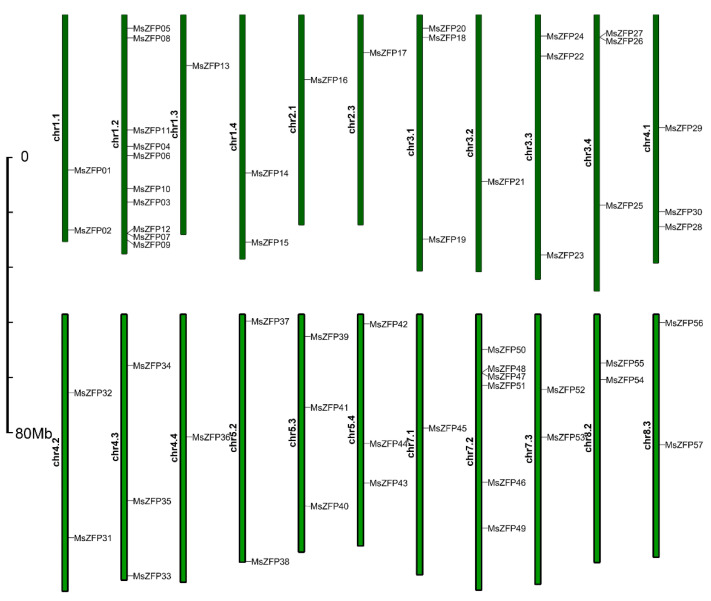
Genomic distributions of 58 *MsZFP* genes across the 32 alfalfa chromosomes. In the “*MsZFP* gene distribution map” of 22 alfalfa chromosomes, the green bars represent each chromosome, and the black lines indicate the position of each *MsZFP* gene.

Based on the alfalfa genome data and chromosomal (Chr.) annotation, all *MsZFP* genes were unevenly distributed on 22 Chr. except *MsZFP58*, which was located on unmapped scaffolds. There were no *MsZFP*s located on Chr. 6. As shown in Figure 3, a total of ten *MsZFP*s were located on Chr. 1.2, which had the largest number of *MsZFP*s. Only one *MsZFP* gene was distributed on chromosomes 1.3, 2.1, 2.3, 3.2, 4.4, and 7.1. In addition, there were two *MsZFP* genes located on chromosomes 1.1, 1.4, 4.2, 5.2, 7.3, 8.2, and chromosome 8.3. Three *MsZFP*s were located on chromosomes 3.1, 3.3, 3.4, 4.1, 4.3, 5.3, and chromosome 5.4.

**Figure 4:**
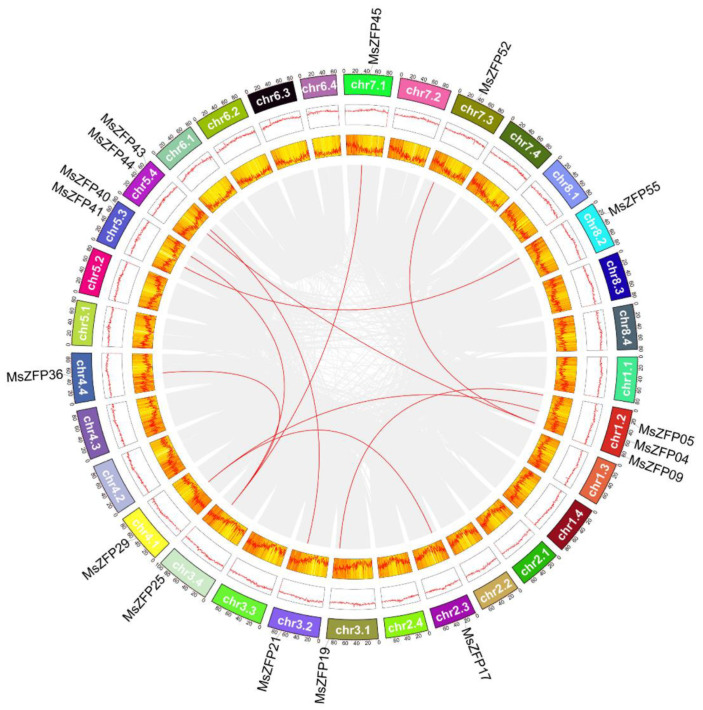
Synteny analysis of *MsZFP* genes in alfalfa. Grey lines represent all synteny blocks in the alfalfa genome. Red lines indicate the duplicated *MsZFP* gene pairs in alfalfa. Yellow and red boxes indicate the gene density and GC content of all genes in alfalfa, respectively.

According to the duplication events of the *MsZFP* gene family, we can analyse the *MsZFP* gene duplication event in alfalfa. The substitution rate of non-synonymous (Ka) and synonymous (Ks) is the basis for assessing the selection pressure of duplication events. A Ka/Ks value of 1, <1, or >1 indicates neutral selection, purification selection, and positive selection, respectively. [10]. TBtools was used to visualize the collinearity relationship between these *MsZFP*s in alfalfa. In this research, a total of ten pairs of *MsZFP* genes (16/58, 27.59%) were confirmed as segmental duplications, which were randomly distributed on twelve chromosomes (Figure 4, Table S6). The Ka/Ks of duplicated *MsZFP*s varied from 0.2482 to 0.9344, which means that these synteny genes evolved under purifying selection.

**Figure 5:**
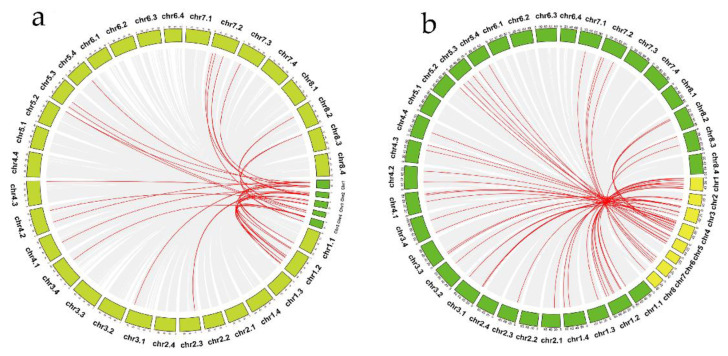
Synteny analysis of *MsZFP* genes with (**a**) *Arabidopsis* and (**b**) *Medicago truncatula*. The red line represents gene pairs that are homologous.

To better understand the evolutionary relationship of these *MsZFP* genes, we selected two model plants, *Arabidopsis* and *M. truncatula*, in order to analyse their synteny relationship. In our research, we identified 29 pairs of orthologues between *Arabidopsis* and *M. sativa* (Figure 5A and Table S7), and the ratio of Ka/Ks varied from 0.1170 to 1.4973. In addition, we identified 42 pairs of orthologues between *M. sativa* and *M. truncatula* with synteny relationships (Figure 5B, Table S8); the ratio of Ka/Ks varied from 0.0453 to 2.4149. Furthermore, comparing the above results, it is easy to conclude that the *M. sativa* genes have higher similarity with *M. truncatula*.

### 3.5. Analysis of MsZFP Gene Promoter Cis-Regulatory Elements

*Cis*-regulatory elements can regulate transcription for precise initiation and efficiency of genes by binding to transcription factors. Therefore, we explored the composition of ten *cis*-regulatory elements (ABREs, AREs, CGTCA motifs, DREs, G-boxes, TGACG motifs, MBSs, LTRs, TC-rich repeats, and TCA elements) in the promoter regions of the 58 induced *MsZFP*s under abiotic stress (Figure S1, Table S9). This study aimed to systematically analyse these regulatory elements, aiming to be assist in the discovery of antistress genes. AREs had the largest number of *cis*-regulatory elements, with 140. However, the DREs only had two *cis*-regulatory elements in these *MsZFP*s. In addition, according to Figure S2, we found that only *MsZFP11* had nine *cis*-regulatory elements, while *MsZFP58* did not contain any of these ten *cis*-regulatory elements.

### 3.6. Expression Patterns of the MsZFPs in Different Tissues

**Figure 6:**
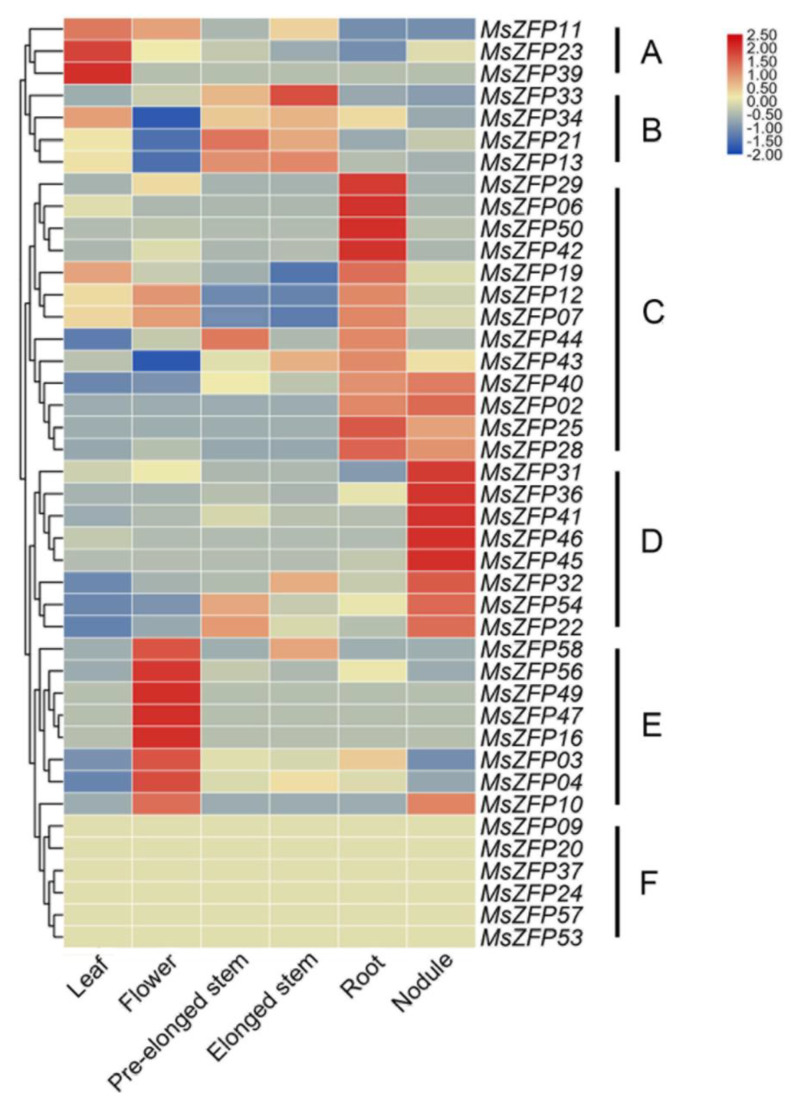
Heatmap representation of the expression patterns of *MsZFP*s among different tissues. Lower and higher levels of transcript accumulation are indicated by red and blue, respectively, and the median level is indicated by light yellow. Microarray data were downloaded from the CADL-Gene Expression Atlas database, and the heatmap was constructed using TBtools. Subgroups A to F displayed the highest transcript accumulation level in different tissues.

Tissue-specific expression is usually related to the specular function of *MsZFP* genes in alfalfa. According to the downloaded alfalfa B47 genotype expression database, we evaluated the transcript abundance pattern of the *MsZFP*-encoding genes in a total of six different tissues: leaf, flower, pre-elongated stem, elongated stem, root, and nodule; 42 *MsZFP* genes were detected in the database. A heatmap was then constructed to visualize these expression patterns with TBtools. According to their expression patterns, 42 *MsZFP*s were clustered into six clades, named A to F (Figure 6). Cluster A contained three genes, which displayed the highest expression level in root tissues. Additionally, subgroups C, D, and E showed high expression levels in root, nodule, and flower tissues, respectively. Clade B included four genes that exhibited variable expression profiles in these six tissues. In contrast, subclade F showe no expression in the six plant tissues.

### 3.7. Expression Analysis of the MsZFPs in Abiotic Stress

To probe the transcript abundance of *MsZFP* genes under cold, drought, salt and ABA stress, we used BLASTP against transcriptome datasets constructed by our laboratory. A total of 57 *MsZFP*s were induced under four stresses; these expression patterns are displayed in Figures S2–S5. For the cold treatment, all 57 induced *MsZFP*s were divided into seven groups based on their expression pattern at five time points. The expression of Group C was highly upregulated at 0–2 h and rapidly declined at the subsequent time points. In contrast, clade F displayed the opposite results, in which expression levels were severely downregulated at 0–2 h and remained relatively stable. Interestingly, subgroup E displayed light expression levels at 2 h and gradually peaked at 24 h. Groups A, B, and D *MsZFP*s showed variable expression profiles at all five time points. Group G showed no expression at any time under cold stress.

For the ABA treatment, all the induced genes were divided into seven categories. Group A was severely and continually downregulated with the extension of treatment. Genes of Groups B and D were highly upregulated at 3 h and were inhibited or unexpressed at other time points. Group C revealed variable expression levels at four time points. The expression levels of clades E, F, and G were slightly upregulated at 3 h and reached their maximum values at 12 h.

Under drought treatment, all these genes were induced at different levels. According to Figure S4, Group A exhibited a process of upregulation and reached its maximum level after drought removal 24 h. The expression levels of Groups C and D fluctuated after treatment and were higher after rehydration than before treatment. Groups E and G were upregulated at the beginning of treatment at 1 h and 24 h, respectively, and gradually downregulated with the extent of processing time, suggesting that the genes of Group E can more rapidly respond to drought stress than the genes of Group G. The expression profile of clade F gradually decreased under the treatment of drought stress from 1 to 24 h, and reached its minimum level after drought removal 12 h, suggesting positive regulation.

According to Figure S5, it is not difficult to speculate that all 57 *MsZFP*s were positive to salt stress, with variable expression levels. The expression of Group A exhibited a dynamic change before removal of salt stress for 12 h and suddenly reached a peak. In addition, the genes of subgroup B were highly sensitive to salt stress and rapidly reduced after treatment for 1 h. Group C reached its maximum minimum level at 3 h and 12 h after removal, respectively. Similar to Group C, Group D reached these two peak values at 6 h and 12 h after removal, respectively. Group D displayed a negative response to salt stress, with the expression level not significantly upregulated until treatment for 6 h, and rapidly reduced after stress removal. Groups F and G were highly sensitive to salt stress; however, their expression levels did not change significantly after stress treatment.

### 3.8. Validation of qRT-PCR

**Figure 7:**
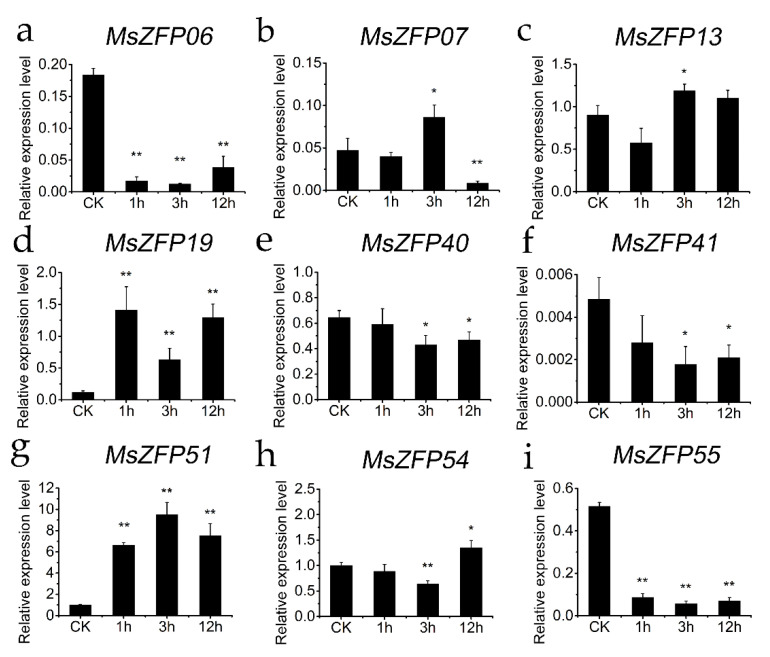
Expression analysis of nine *MsZFP* genes under ABA treatment, revealed by qRT-PCR. Values represent the average ± SD of root tips. Error bars represent the standard errors of the means (*n* = 3). The relative expression level was normalized according to the values in the control (0 h). And a to i means the results of qRT-PCR of selected *MsZFP*s “*” indicates significance at the 0.05 level, and “**” indicates significance at the 0.01 level.

Using the RNA-seq data on abiotic stress, qRT–PCR was performed to validate the nine *MsZFP*s that were significantly induced (Figures S3–S7), under drought, salt stress and ABA treatment; their primer sequences for qRT-PCR are listed in Table S1. Based on the qRT-PCR analysis, the selected *MsZFP* genes were induced to varying degrees by different stresses. Notably, most of these *MsZFP* genes were significantly upregulated under all abiotic stresses, suggesting positive regulation. At the same time, the expression trend of the selected Q-type C2H2 genes was consistent with the RNA-seq analysis results. For example, *MsZFP19*, and *MsZFP51*, whose expression levels were rapidly upregulated with time under ABA treatment, showed results similar to the RNA-seq data (Figure 7). Interestingly, the variation trend of the expression of *MsZFP41* under ABA treatment initially decreased and then increased, which was different from the variation trend of the expression of *MsZFP41* under drought treatment.

## 4. Discussion

Q-Type ZFPs are a subfamily of C2H2-ZFPs, which play crucial roles in plant response to abiotic stress. Previous studies have identified many Q-type C2H2 ZFPs in plants such as *Arabidopsis*, rice, wheat, poplar, and potato [9,10,36,37,38]. However, there have been few studies on the molecular function of *MsZFP*s under abiotic stress conditions in alfalfa. Therefore, this research aimed to analyse this gene family in depth, hoping to be assist in the mining of antistress genes. In this research, we investigated the features of Q-type C2H2 ZFPs using previous studies of other plants; a total of 58 Q-type *MsZFP*s were identified through genome-wide identification. Then, the physical and chemical properties, gene structure, conserved motifs, phylogenetic relationships, chromosomal mapping, synteny analysis, *cis*-regulatory element composition, specific expression of tissues and expression pattern under abiotic stress were all investigated. The results provided significant and valuable information to aid in better exploring the evolutionary relationship and function of these *MsZFP*s.

In our research, 58 Q-type C2H2 ZFPs were identified in alfalfa, more than were identified in *Triticum aestivum* [39], but fewer than the number identified in potato [10] and rice [40]. The lengths of these protein sequences were between 126 and 1633 amino acids (Table S3). Phylogenetic analysis showed that 58 *MsZFP*s were divided into 12 groups, which were similar to *StZFP*s in *Solanum tuberosum* L. [10]. Moreover, some of the selected *Arabidopsis* Q-type C2H2 ZFP genes and the *MsZFP*s were clustered into the same clade, suggesting that they may have similar functions under abiotic stress and a close evolutionary relationship. Changes in their intron–exon numbers can affect the function of genes during evolution, and previous studies have shown that the fewer introns the genes possess, the easier the function of genes during environmental challenge [41]. In this study, all the *MsZFP*s were divided into six groups, and most of the *MsZFP*s were intron-less. In addition, motif 1 was widely distributed in *MsZFP*s. Notably, motif 5 was usually located at the end of these proteins, and among them, 56 (56/58, 96.55%) *MsZFP* genes had at least the “LxLxL” type of EAR motif, suggesting that the *MsZFP*s are rich in potential transcriptional inhibitors. In addition, previous research has identified many transcriptional repressors in plants [42]. For example, the *LATE FLOWERING* (*LATE*) gene in *Arabidopsis* [43] and the *KNUCKLES* (*KNU*) [44], a transcriptional repressor of cellular proliferation in *Arabidopsis*, were clustered in the same subclade as *MsZFP51* and *58*, suggesting that these two *MsZFP*s have functions similar to transcriptional repressors.

Chromosomal mapping analysis showed that all Q-type *MsZFP* genes were dispersed on 22 chromosomes. In addition, all of the copies of chromosomes 1, 3, and 4 (*M. sativa* is an autotetraploid plant, and each chromosome has four copies) with Q-type *MsZFP*s were caused by its complex genetic background. Gene duplication events usually contribute to the emergence of members of gene families as new functions are produced in plant genome evolution [45]. In our study, a total of ten pairs of *MsZFP* genes (16/58, 27.59%) were identified as segmental duplications, and most syntenic *MsZFP*s were located in the middle of chromosomes. *MsZFP25* is collinear with three genes, and all four genes are clustered in the C3 group, suggesting that they may have a similar origin. Therefore, it is important to investigate their duplication relationship in order to further research on the evolutionary relationship of this gene family.

*Cis*-regulatory elements are located upstream of genes and influence gene functions by binding with TFs. In the current study, the promoter regions of *MsZFP*s had many response elements, mainly hormone-related elements, light-related elements and stress response elements. Among these, AREs were most widely present in the *MsZFP*s, followed by ABREs and G-boxes. In addition, only *MsZFP11* and *MsZFP13* had DRE regulatory elements, while *MsZFP58* did not have any *cis*-regulatory elements among the investigated regulatory elements. A previous study confirmed that many important *cis*-regulatory elements already existed in the promoter regions of stress response genes in MYBs, WRKYs, and MADS-boxes [35,40,46]. Furthermore, many of these elements, such as ABREs and DREs, have been reported to widely participate in abiotic stress responses in *Artemisia annua*, *Arabidopsis* and wheat [47,48,49]. However, no study has investigated these key regulatory elements in alfalfa. Therefore, according to these results, which provide many candidate genes for anti-abiotic stress, their specific functions need to be confirmed by further study.

Specific expression of *MsZFP* genes in different tissues could provide a clear study direction for plant growth development. Gene expression patterns can provide significant supportive data to better understand the function of *MsZFP*s. According to Figure 6, a total of 36 *MsZFP*s were expressed in different tissues to variable degrees; each group displayed tissue-specific expression patterns, especially in Groups C, D and E, which showed higher expression levels in roots, nodules, and flowers, respectively. Therefore, we can speculate that these may become the key regulatory TFs in regulating plant growth and flowering According to these tissue-specific expression patterns, *MsZFP* genes widely participate in the process of growth development with the related tissue, which provides insight into how we can promote special tissue growth and development by changing the expression level of these *MsZFP*s using biotechnology.

Environmental stresses can affect the growth of plants and crop productivity [50]. A previous study explored many *C2H2-ZFP*s, which are widely related to abiotic stress responses, in various plants such as *Arabidopsis* [51], petunia [52], and potato [53]. For example, *ZAT10* is a transcriptional repressor in *Arabidopsis*, and overexpression of *ZAT10* can enhance the tolerance of plants to abiotic stress [54]. The expression level of *StZFP1* in potato can be induced by salt stress, drought stress and exogenously applied ABA, and ectopic expression of *StZFP1* in tobacco can increase tolerance to salt [53]. In *Oryza sativa*, *ZFP179* has been implicated in plant development and stress responses induced by salt, drought, and ABA treatments at the seedling stage. Overexpression of *ZFP179* in rice can increase salt tolerance and shows high sensitivity to exogenous ABA in seedlings [36]. In our study, a total of 57 *MsZFP* expression patterns were explored using RNA-Seq data previous obtained in our laboratory. Most of these *MsZFP*s were induced under different abiotic stresses. According to the expression profiles in Figures S2–S5, we found that *MsZFP20*, *MsZFP26*, *MsZFP37*, *MsZFP42*, *MsZFP44*, *MsZFP51*, and *MsZFP58* were induced under ABA, salt, and drought stress. However, these genes were negatively associated with cold stress in alfalfa. In the current study, we verified the expression levels of nine *MsZFP*s under abiotic stress using qRT-PCR. However, some of the fold changes between the RNA-Seq data and qRT-PCR results were different, which was also found in prior research [40,55,56] and may be caused by the difference in genetic diversity among alfalfa individuals, or by differences in sampling time [57]. In conclusion, the expression patterns of *MsZFP* genes under various abiotic stresses will provide many new candidate genes for further exploration of the mechanisms of resistance in alfalfa.

## 5. Conclusions

In the current research, we provided a comprehensive analysis of 58 Q-type *C2H2-ZFP*s in the alfalfa genome. These *MsZFP*s were divided into twelve groups, with most of the *MsZFP*s closely related to known antistress genes in *Arabidopsis*. We explored their physiochemical properties, phylogenetic relationships, gene structures, motif composition, and *cis*-regulatory elements. Chromosomal mapping and collinearity analysis revealed the duplication relationship of alfalfa *MsZFP*s. In addition, the tissue expression patterns showed that many of the *MsZFP*s were expressed in specific tissues at different levels. We selected nine candidate genes that were highly induced under abiotic stress and used qRT-PCR to validate their expression patterns. Our study contributes to understanding of the roles of *MsZFP* genes under abiotic stress and provides resources for further molecular and functional analysis in alfalfa. It may also contribute to the improvement of abiotic stress tolerance using molecular biology techniques such as RNA interference, overexpression, and CRISPR/Cas9 technology in future research.

## Data Availability

The draft genome data of alfalfa was obtainedfrom https://figshare.com/projects/whole_genome_sequencing_and_assembly_of_Medicago_sativa/66380, accessed on 23 May 2021. The *Arabidopsis* and *M. truncatula* C2H2 protein sequences were downloaded from The *Arabidopsis* Information Resource (TAIR9) (www.arabidopsis.org, accessed on 26 May 2021) and the published article, respectively. Genome-wide transcriptome data of different alfalfa tissues were acquired from the CADL-Gene Expression Atlas (https://www.alfalfatoolbox.org, accessed on 28 May 2021). All transcriptome sequencing data used in this study are available in NCBI SRA (https://www.ncbi.nlm.nih.gov/sra/, accessed on 29 May 2021): SRR7091780-SRR7091794 (cold treatment) and SRR7160313-SRR7160357 (ABA, drought and salt treatments).

## Supplementary Materials

The following are available online at https://www.mdpi.com/article/10.3390/genes12121906/s1, Additional file 1: Table S1. Information of the selected *MsZFP* primers used in quantitative real-Time PCR. Table S2. The putative58 *MsZFP* protein sequences in *Medicago sativa* L. Table S3. Summary of the Q-type C2H2 transcription factor gene in alfalfa. Table S4. The putative 58 Q-type C2H2 ZFP protein sequences in *Arabidopsis thalia*. Table S5. MEME motif sequences in predicted MsZFP proteins. Table S6. Ka/Ks ratios of the syntenic relationships between *MsZFP*s. Table S7. Ka/Ks ratios of the syntenic relationships between alfalfa and *Arabidopsis*. Table S8. Ka/Ks ratios of the syntenic relationships between alfalfa and *Medicago truncatula*. Table S9. The description of the selected *cis*-regulatory elements. Additional file 2: Figure S1. Analysis of *cis*-regulatory elements of the promoter regions of *MsZFP* genes of alfalfa. Figure S2. Expression of 57 *MsZFP* genes in response to cold treatment. Figure S3. Expression of 57 *MsZFP* genes in response to ABA treatment. Figure S4. Expression of 57 *MsZFP* genes in response to drought stress. Figure S5. Expression of 57 *MsZFP* genes in response to salt stress. Figure S6. Expression analysis of nine genes under drought treatment, revealed by qRT-PCR. Figure S7. Expression analysis of nine *MsZFP* genes under salt treatment, revealed by qRT-PCR.
