# Supplementary material for: Genome-Wide Identification of the Q-type C2H2 Transcription Factor Family in Alfalfa (Medicago sativa) and Expression Analysis under Different Abiotic Stresses
Source: Genes (Basel). 2021 Nov 27;12(12):1906. doi: 10.3390/genes12121906 (PMC8701282; doi:10.3390/genes12121906)
Supplement: Supplementary file 1 [file genes-12-01906-s001.zip › genes-1478807-supplementary/Additional file 2.pdf]

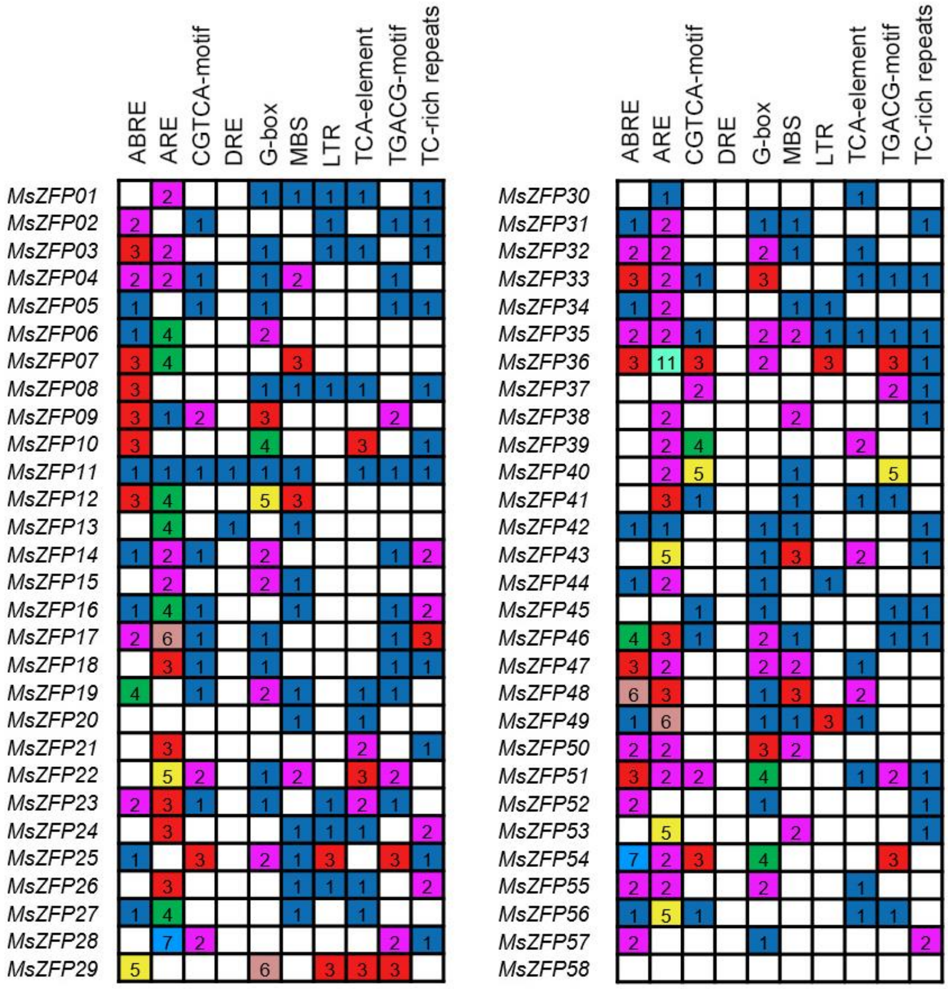

Figure S1. Analysis of *cis*-regulatory elements of the promoter regions of *MsZFP* genes of alfalfa. The differently coloured boxes represent the different regulatory elements, and the numbers indicate the numbers of *cis*-regulatory elements in the promoter regions of *MsZFP* genes.

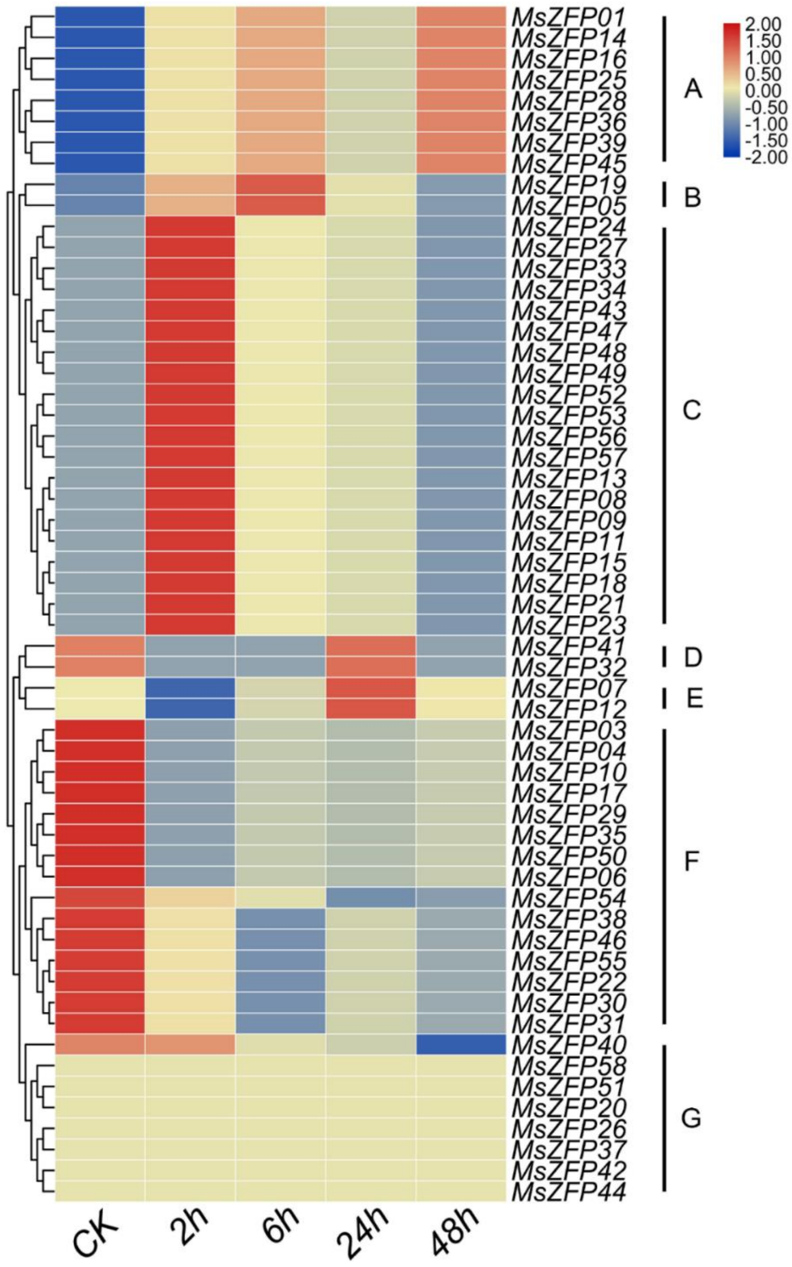

Figure S2. Expression of 57 *MsZFP* genes in response to cold treatment. Heatmap displaying the changes in the expression level of these *MsZFP* genes at different time points after treatment at 4 °C (0, 2, 6 24, and 48 h) in the whole seedling, where "CK" indicates 0 h. Microarray data were obtained from the reported study in alfalfa. The *MsZFP*s of subgroup F were inhibited during cold stress, and members of subgroup G were not induced under cold stress.

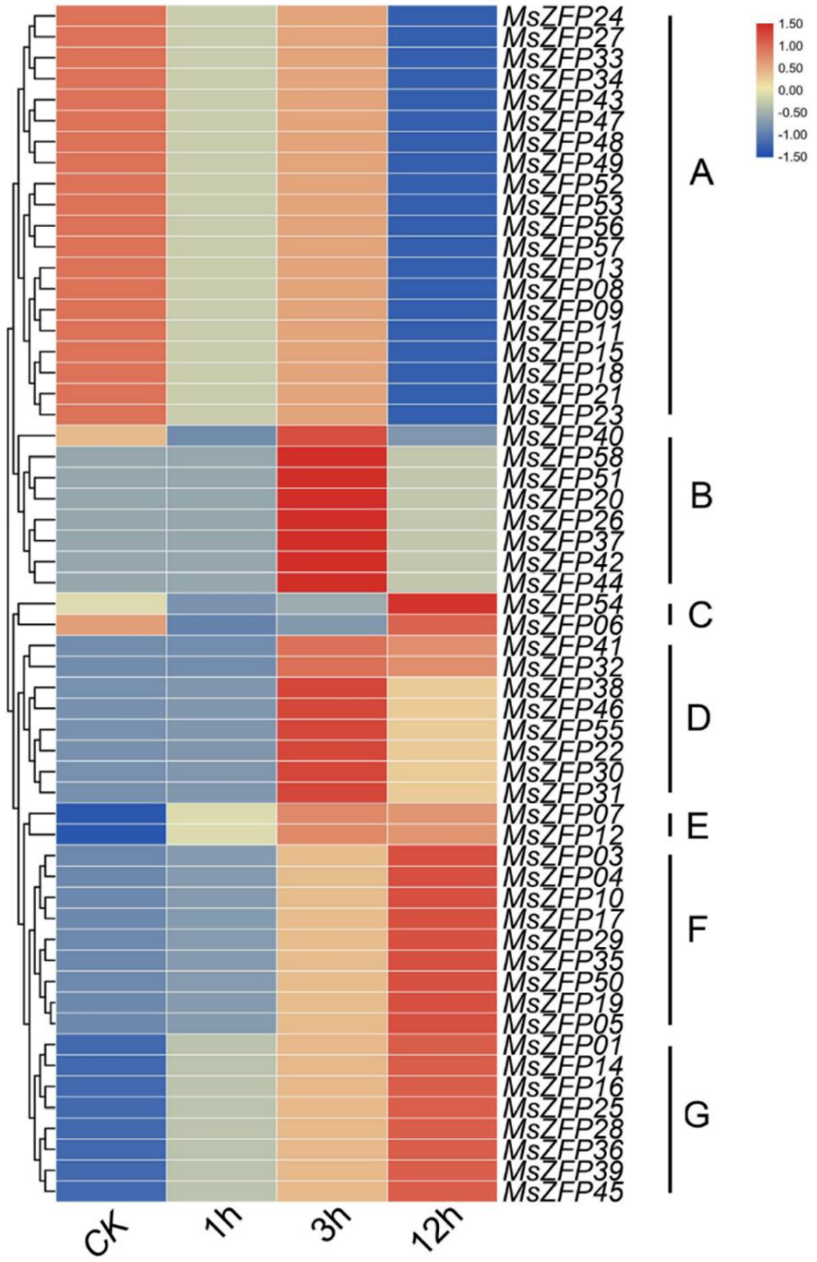

Figure S3. Expression of 57 *MsZFP* genes in response to ABA treatment. Heatmap displaying the changes in the expression level of these *MsZFP* genes at different time points after treatment with 10 μM abscisic acid (0, 1, 3, and 12 h) in the root tip, where "CK" indicates 0 h. Microarray data were obtained from the reported study in alfalfa.

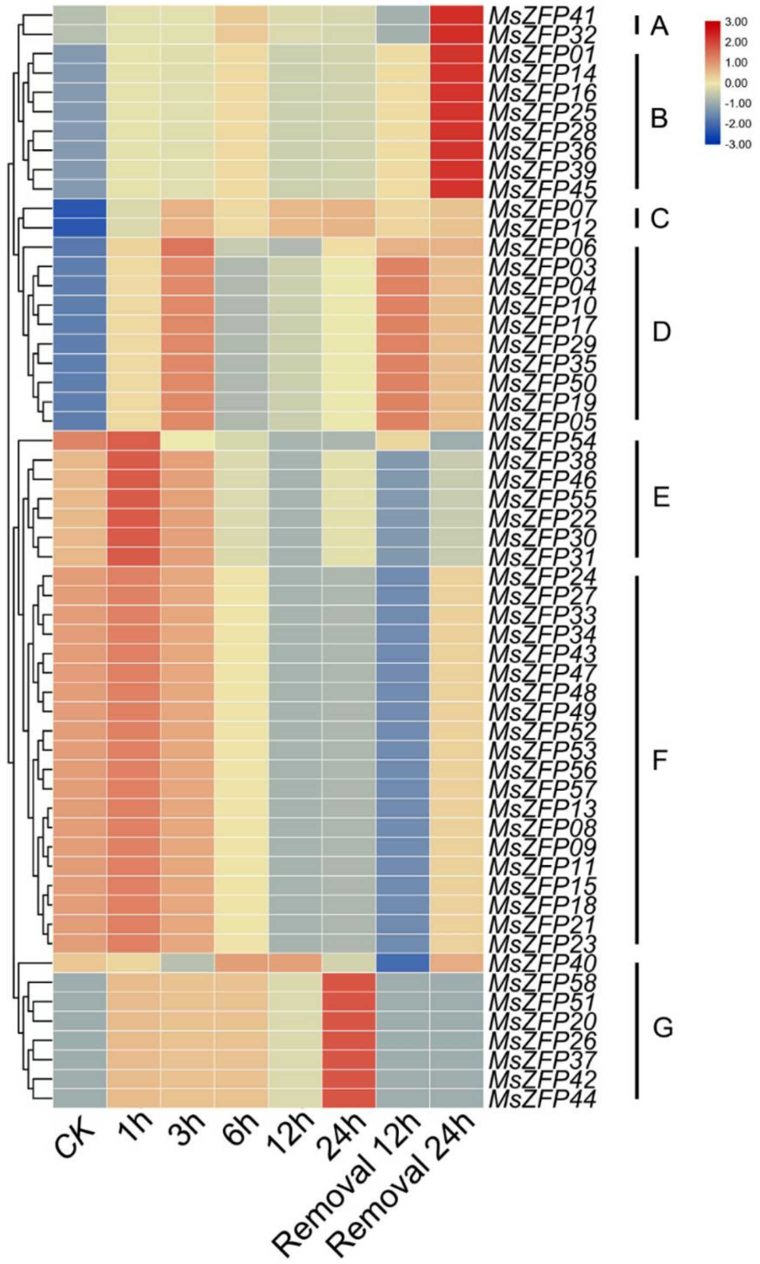

Figure S4. Expression of 57 *MsZFP* genes in response to drought stress. Heatmap displaying the changes expression level of these *MsZFP* genes at different time points after treatment with 400 mM mannitol (0, 1, 3, 6, 12, 24, removal 1 and 12 h) in the root tip, and "CK" indicates 0 h. Microarray data were obtained from the reported study in alfalfa. The *MsZFP*s of subgroup F were inhibited during drought stress, and members of the other subgroups were induced under drought stress.

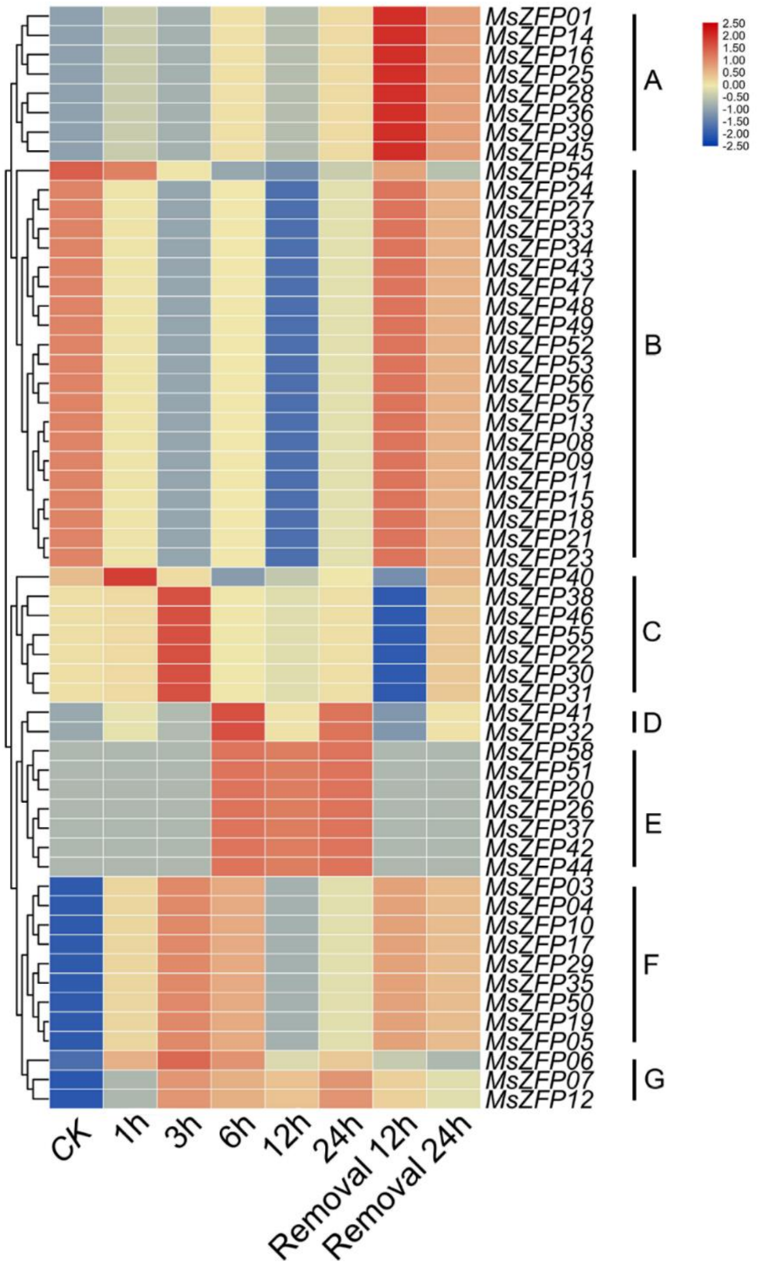

Figure S5. Expression of 57 *MsZFP* genes in response to salt stress. Heatmap displaying the changes expression level of these *MsZFP* genes at different time points after treatment with 250 mM NaCl (0, 1, 3, 6, 12, 24, removal 1 and 12 h) in the root tip, and “CK” indicates 0 h. Microarray data were obtained from the reported study in alfalfa.

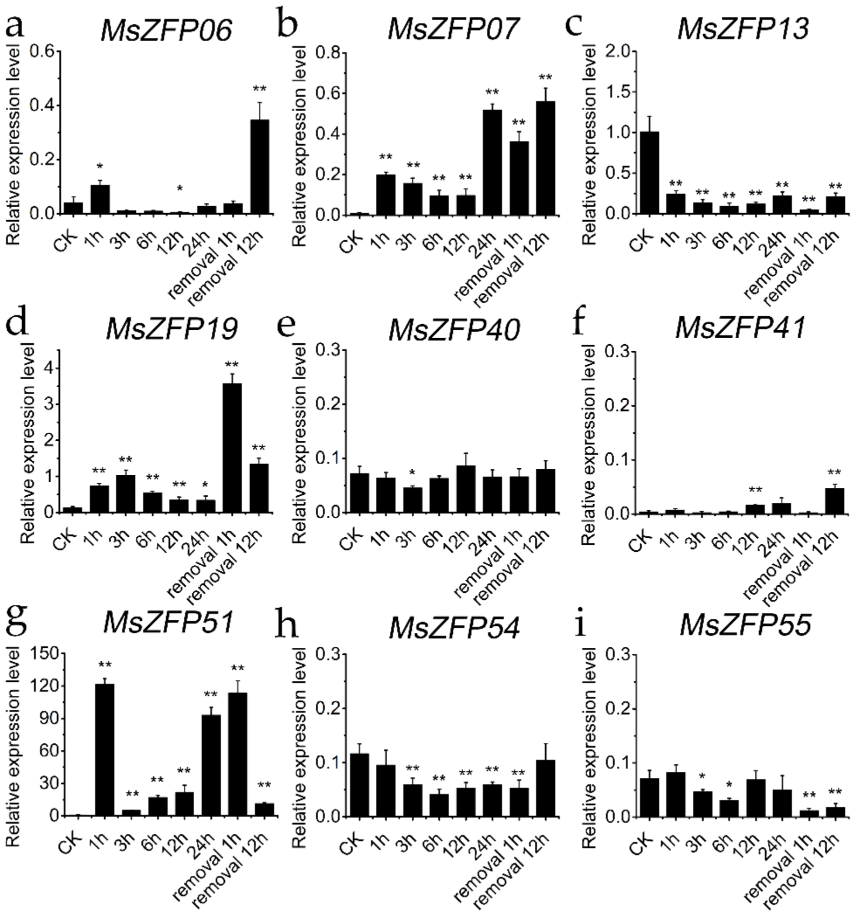

Figure S6. Expression analysis of nine genes under drought treatment, revealed by qRT-PCR. Values represent the average ± SD of root tips with three technical replicates under cold treatment. Error bars represent the standard errors of the means (n=3). The relative expression level was normalized according to the values in the control (0 h). "*" indicates significance at the 0.05 level, and "**" indicates significance at the 0.01 level.

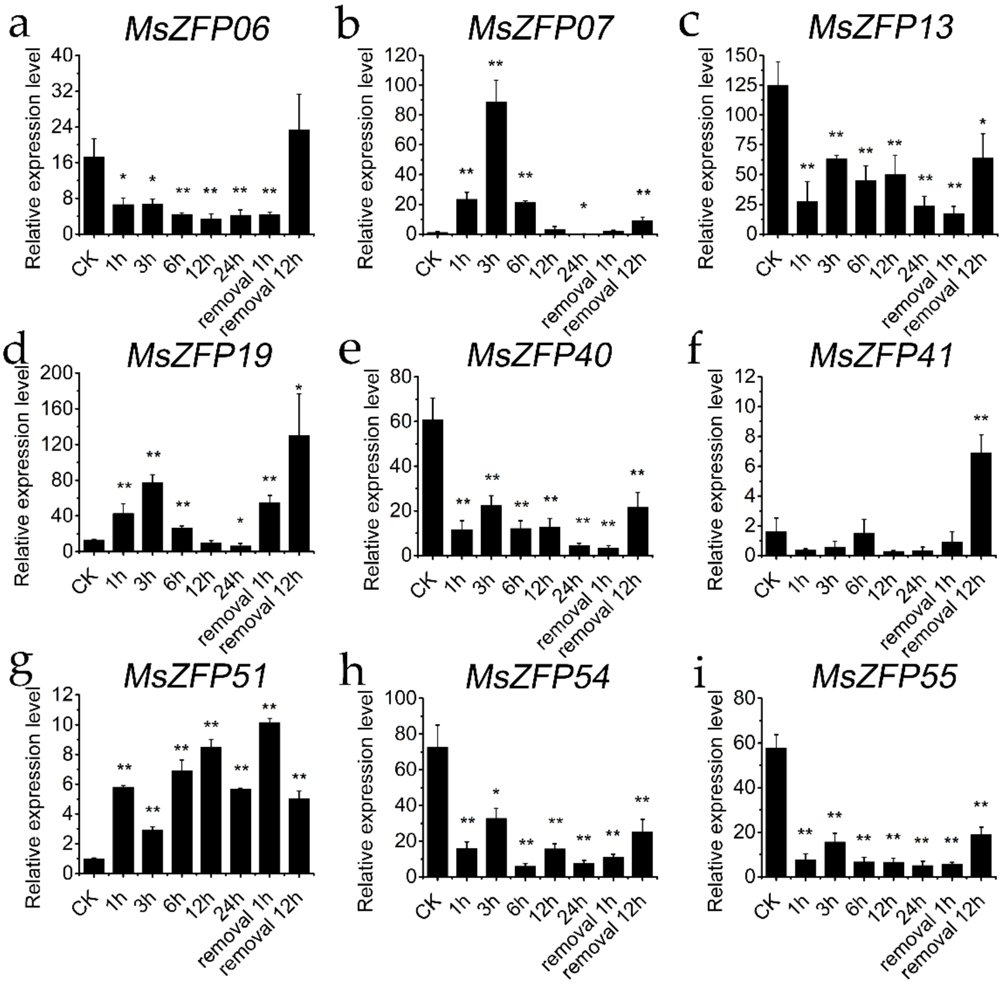

Figure S7. Expression analysis of nine *MsZFP* genes under salt treatment, revealed by qRT-PCR. Values represent the average ± SD of root tips. Error bars represent the standard errors of the means (n = 3). The relative expression level was normalized according to the values in the control (0 h). "*" indicates significance at the 0.05 level, and "**" indicates significance at the 0.01 level.
